# A Diffusion Model Analysis of Magnitude Comparison in Children with and without Dyscalculia: Care of Response and Ability Are Related to Both Mathematical Achievement and Stimuli

**DOI:** 10.3389/fpsyg.2017.01615

**Published:** 2018-01-12

**Authors:** Carsten Szardenings, Jörg-Tobias Kuhn, Jochen Ranger, Heinz Holling

**Affiliations:** ^1^Department of Psychology, Westfälische Wilhelms-Universität Münster, Münster, Germany; ^2^Department of Psychology, Martin-Luther-Universität Halle-Wittenberg, Halle, Germany

**Keywords:** dyscalculia, diffusion model, approximate number system, access deficit, magnitude comparison, dot set comparison, response caution, mathematics anxiety

## Abstract

The respective roles of the approximate number system (ANS) and an access deficit (AD) in developmental dyscalculia (DD) are not well-known. Most studies rely on response times (RTs) or accuracy (error rates) separately. We analyzed the results of two samples of elementary school children in symbolic magnitude comparison (MC) and non-symbolic MC using a diffusion model. This approach uses the joint distribution of both RTs and accuracy in order to synthesize measures closer to ability and response caution or response conservatism. The latter can be understood in the context of the speed-accuracy tradeoff: It expresses how much a subject trades in speed for improved accuracy. We found significant effects of DD on both ability (negative) and response caution (positive) in MC tasks and a negative interaction of DD with symbolic task material on ability. These results support that DD subjects suffer from both an impaired ANS and an AD and in particular support that slower RTs of children with DD are indeed related to impaired processing of numerical information. An interaction effect of symbolic task material and DD (low mathematical ability) on response caution could not be refuted. However, in a sample more representative of the general population we found a negative association of mathematical ability and response caution in symbolic but not in non-symbolic task material. The observed differences in response behavior highlight the importance of accounting for response caution in the analysis of MC tasks. The results as a whole present a good example of the benefits of a diffusion model analysis.

Developmental dyscalculia (DD) is a specific learning disorder that affects the acquisition of arithmetic facts (Landerl et al., 2004), arithmetic skills and number processing in children (Kuhn, 2015). Though terminology and definition vary slightly among authors, most definitions of DD entail low mathematical achievement that can not be explained by inadequate schooling, low intelligence, or age. Hence in this paper we mean by DD a score of 85 or lower in an age-appropriate standardized mathematical achievement test and score higher than 85 in a standardized age-appropriate intelligence test and no prolonged interruptions of school education.

In an academic context, children affected by DD display longer solution times, higher error rates, and use immature solution strategies (for example counting) for calculations (Geary et al., 1992). Past research results include longer response times (RT) and lower accuracy, i.e., higher error rate, in magnitude comparisons (De Smedt et al., 2013; Schneider et al., 2016).

Despite a large body of results, the causal mechanisms of DD are not fully understood. Amongst others (see Landerl et al., 2004; Andersson and Östergren, 2012 for an overview) there are two possible explanations:

The Approximate Number System (ANS) (see for example Feigenson et al., 2004) is a system innate in humans and many animals that allows for a quick approximation for the number of objects. Even before the term ANS was used, Dehaene and Changeux (1993) suggested that such a system forms the basis of arithmetical skills. A connection between the ANS and mathematical performance (or DD in particular) was for example found by Feigenson et al. (2004) and more recently by Mazzocco et al. (2011).

On the other hand, Rousselle and Noël (2007) could only find a connection between mathematical ability and the performance in numeral comparison. They suggested that not an impaired ANS causes DD but a deficient access to magnitude representation in the ANS from numerals, in short access deficit (AD). Rousselle and Noël (2007) assume, that in order to compare numerals regarding their associated magnitudes these numerals have to be mapped to a cognitive representation of associated magnitudes first and then these representations are compared. We will stick to this model for the remainder of the introduction. As this assumption is debatable (De Smedt et al., 2013), we will interpret our findings in other contexts as well.

In this study we investigate to which extent an impaired ANS or an AD underlie DD by using a diffusion model. The next subsection elaborates why analyses of MC data based on RT or accuracy alone are insufficient to answer this question, the subsection thereafter gives a brief introduction to diffusion models and relates their parameters to ANS and AD.

## Past research and its limitations regarding the role of the approximate number system and an access deficit in dyscalculia

**Figure 1:**
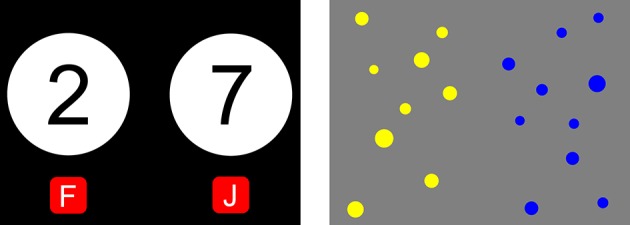
Screenshots of symbolic **(Left)** and non-symbolic **(Right)** MC. In both tasks a subject has to decide which one out of two magnitudes (given by numerals or number of dots) is larger.

Magnitude comparison (MC) is a frequently used task paradigm in DD research. In MC tasks subjects judge which of two simultaneously displayed magnitudes is larger. The magnitudes are either represented by numerals (symbolic MC; Moyer and Landauer, 1967) or sets of dots (non-symbolic MC; Halberda et al., 2008). Sample stimuli of both types are shown in Figure 1. Sometimes only one magnitude is presented (as numeral or set of dots) which has to be compared to a priorly memorized reference magnitude (see for example Ratcliff et al., 2012, 2015). For a detailed introduction to MC see De Smedt et al. (2013) and Kuhn (2015).

Suppose a subject solves a symbolic MC (see Figure 1). The image first has to be encoded into numerals, the numerals have to be connected to their corresponding magnitudes, the magnitudes will be compared, and finally a response corresponding to the outcome of the comparison will be executed. Thus an impairment of the ANS or an AD would affect the ability for symbolic and non-symbolic MC differently:

1. An impaired ANS would affect both types of MC equally, since an impaired ANS implies an impaired ability to compare magnitudes, which is needed equally regardless of the mode of presentation.
2. AD would affect symbolic MC, but not non-symbolic MC, since in the latter no magnitude information has to be accessed from numerals.

If both hypotheses were true, it would affect both MC types but more so symbolic MC, since in the latter two processes introduce errors and slowdown, where in non-symbolic MC only one such source exists. Indeed in a meta-analysis Schneider et al. (2016) found a significantly stronger correlation between RT in symbolic MC and mathematical achievement than between the latter and RT in non-symbolic MC. On the other hand, correlation of accuracy with mathematical achievement did not differ significantly between symbolic and non-symbolic MC. In order to draw conclusions regarding ANS or AD from these findings one has to assume that RT or accuracy is a measure of ability. After all, AD and an impaired ANS only have implications regarding ability. However, any such assumption is not straightforward:

Suppose there are two subjects solving symbolic MC that only differ in their ability to compare magnitudes, for example due to differences in the ANS, and they are equal in every other way related to the task. The subject with lower ability either needs more time for the comparison or makes more errors or both. So when recording RT and accuracy for these two subjects in several symbolic MC tasks, we expect to see either a higher mean (or median) RT or a lower mean accuracy or both.

Now suppose there are two subjects solving symbolic MC that only differ in their response behavior, in particular their response caution, but are equal in every other way related to the task. The subject with higher response caution takes more time and commits less errors in return (that is in case there are any errors). So when recording RTs and accuracy for these two subjects in several symbolic MC tasks we expect to see a higher mean (or median) RT and higher mean accuracy.

As a conclusion, if one only looks at RTs, one cannot distinguish a lower ability from higher response caution, and if one looks only at accuracy, one cannot distinguish a lower ability from lower response caution. As response caution varies between people and situations, aggregating results over people and studies and inferring high or low ability from that data is problematic in general.

## The diffusion model

A cognitive process model that allows to separate ability and response caution is the diffusion model (DM) that has been introduced into psychology by Ratcliff (1978) as a model for responses and RTs in a timed binary decision task. DMs have been successfully applied to MC data by Park and Starns (2015), Ratcliff et al. (2012), and Ratcliff et al. (2015). They are useful whenever one is interested in the underlying mechanisms of differences rather than just the differences themselves. Ratcliff et al. (2006) could show for example that a difference in performance between young and older adults, which was previously attributed to general slowdown of cognitive processes, could be better explained by just a longer motor reaction time and higher response caution. A DM allows this by assuming an underlying decision making process, the diffusion process, which is thought of as a noisy accumulation of evidence in favor or against an alternative over time. The evidence is thought to lie on a one dimensional metric scale, that is to be a number.

**Figure 2:**
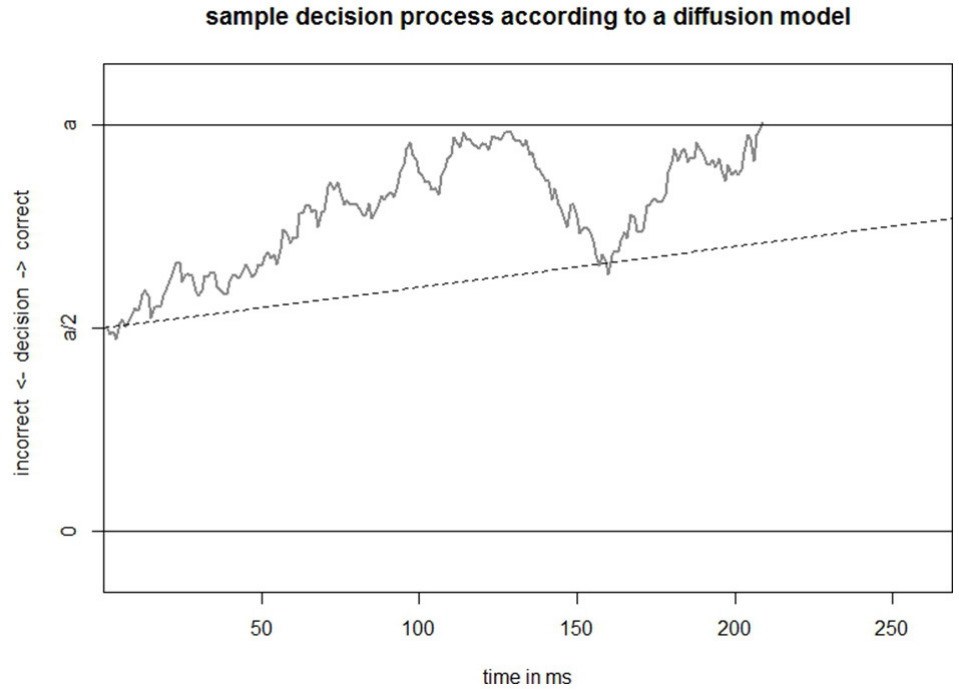
Example of accumulation of evidence (gray) toward the thresholds (solid horizontal lines) with an unbiased starting point according to a DM. Underlying theoretical mean drift is represented by a dashed line.

Figure 2 depicts an example for evidence accumulation over time according to a DM. The evidence toward one alternative at a given time is given by the location on the *y*-axis while the time point is given by the location on the *x*-axis. The solid horizontal lines in Figure 2 represent two thresholds, one corresponding to each possible answer (here in favor or against an alternative). To stay in the context of MCs, suppose the upper threshold corresponds to the correct alternative, say “the larger magnitude is shown on the left hand side,” while the lower threshold corresponds to the incorrect alternative “the larger magnitude is shown on the right hand side.” Once the amount of accumulated evidence is lower than the lower threshold, the process stops and the subject has reached the conclusion that the larger magnitude is shown on the right hand side. Similarly the process stops once the accumulated evidence is higher than the upper threshold but the subject has reached the conclusion that the larger magnitude is shown on the left hand side.

The accumulation of evidence (gray in Figure 2) can be decomposed into an deterministic component, the average amount of collected evidence (dashed line in Figure 2), and a random fluctuation around 0. The average rate of evidence accumulation is called drift rate (*v*) and depends on the subject and the item. We assume that prior to the decision process a subject does not favor one alternative over the other. This is modeled by letting the decision process start equidistantly from both thresholds as depicted in Figure 2.

We put Figure 2 in the context of MC: After a subject has seen a stimulus, say the numerals 4 and 7, the subject encodes it in such a way that a magnitude comparison is possible. Now the decision process begins. Suppose the upper threshold, i.e., the solid horizontal line in Figure 2, corresponds to the correct alternative, 7 > 4, and the lower threshold to the incorrect one, 4 > 7. The decision process starts on the left in between the thresholds and follows on average (for the same item and subject) the trajectory of the dotted line. The slope of the dotted line depends on the ability of the subject, i.e., the rate at which the subject can process information from magnitude representations and the magnitude representations themselves, whose precision depends on the precision of her or his ANS, but in our example it also depends on how well the subject encodes the numeral information into a magnitude representation. Additionally the magnitude representations and thus the slope of the dotted line varies between different stimuli, that is between items. A comparison of 6 vs. 7 would yield a comparatively smaller slope whereas 1 vs. 7 yields a larger slope.

With the assumption of no bias, the outcome of a diffusion process, i.e., the response and the time spent, are determined by randomness, drift rate (*v*), and the distance between the two thresholds called boundary separation (*a*). Note that thus far we have not talked about time that passes outside the decision process, e.g., stimulus encoding and response execution, this time is subsumed in a parameter called non-decision time. For a thorough introduction to DMs we refer the reader to Voss et al. (2013).

Before stating the relation of parameters *a* and *v* to response and RT by Equations (4) and (5), we want to summarize the role of *a* and *v* in non-technical terms:

- If the boundary separation, *a*, increases, both response accuracy as well as RTs will increase; hence this parameter is commonly interpreted as response caution or response conservatism (Voss et al., 2013).
- If the mean drift rate, *v*, increases, response accuracy will increase and RTs will decrease; hence this parameter is commonly interpreted as ability or performance (Voss et al., 2013).

Keeping these interpretations in mind, we can rephrase our previously stated implications of an impaired ANS and AD to statements about *v* in both types of MC:

1. If an impairment of ANS underlies DD, then there will be a negative main effect of DD (relative to healthy controls) on *v* in both types of MC.
2. If AD underlies DD, then there will be negative interaction effect of task type (symbolic relative to non-symbolic MC) and DD (relative to healthy controls) on *v*.

We will test for these effects on *v* in a two-way ANOVA. Park and Starns (2015) found a positive correlation of mathematical performance and *v* in non-symbolic MC, such that we expect to find a negative main effect of DD on *v*. The interaction effect of DD and task type or more generally the relation of mathematical performance or DD to *v* in symbolic MC has not been studied so far. Our study is the first to test the hypothesis of AD using a variable closer to ability than RT and accuracy.

Boundary separation, *a*, in MC has not been looked at except by Park and Starns (2015), who found no association between mathematical performance and *a* in non-symbolic MC. If their results generalized to DD subjects and symbolic MC, we would indeed find no main or interaction effects of DD on *a*. However, it is not implausible that children with a learning disorder respond more cautiously in a test situation; in particular children with DD respond more cautiously when facing numbers. Hence we will also test for effects of DD on *a* in a two-way ANOVA (analogously to effects on *v*). A summary of implicated effects on *a*, *v*, and classical measures (RT and accuracy) is given by Table 1.

**Table 1:** Expected changes in measures depending on source in symbolic and non-symbolic MC: Impaired ANS (ANS), access deficit (AD), and high(er) response caution (HRC).

	**Symbolic MC**				**Non-symbolic MC**			
	**RT**	**Acc**	***v***	***a***	**RT**	**Acc**	***v***	***a***
ANS	+	−	−	°	+	−	−	°
AD	+	−	−	°	°	°	°	°
HRC	+	+	°	+	+	+	°	+

*Direction of the effect is indicated by + and −; null effects are denoted by °*.

In the remainder of this section we will account for the more technical details on DMs in general and the specific variant, that we used.

RTs in a DM are split up in *T*_*d*_, time spent on the decision process, that is the amount of time needed to reach a threshold in Figure 2, and non-decision time, *T*_*er*_, which subsumes all time spent outside the actual decision process, for example encoding the stimulus or executing a motor response. We have the following equation for the total RT (*T*), decision time (*T*_*d*_), and non-decision time (*T*_*er*_):

(1) $$\begin{array}{r} T=T_{d}+T_{er} \end{array}$$

An equation for the expected decision time (*E*(*T*_*d*_)) is given by van der Maas et al. (2011):

(2) $$\begin{array}{r} E\left(T_{d}\right)=\frac{a}{2v}\frac{1-e^{av}}{1+e^{av}} \end{array}$$

*T*_*er*_ varies uniformly between trials around a person specific mean *t*_0_ with a person specific range of *s*_*t*_0__, that is

(3) $$\begin{array}{r} T_{er}\sim U\left(t_{0}-\frac{s_{t_{0}}}{2},t_{0}+\frac{s_{t_{0}}}{2}\right) \end{array}$$

Hence by Equations (1–3) we obtain the following equation for the expected RT (*E*(*T*)) :

(4) $$\begin{array}{r} E\left(T\right)=\frac{a}{2v}\frac{1-e^{av}}{1+e^{av}}+t_{0} \end{array}$$

If *t*_0_ increases, RTs will increase, but the variability of RTs and accuracy is unaffected. A higher *s*_*t*_0__ means higher variability in RTs with everything else being unaffected.

For the sake of completeness we state the probability *P* of a correct response in an unbiased diffusion process with boundary separation *a* and mean drift rate *v*, which is given by

(5) $$\begin{array}{r} P\left(X=1\right)=\frac{e^{av}}{1+e^{av}} \end{array}$$

where *X* denotes the response which is either 0, incorrect, or 1, correct (van der Maas et al., 2011).

There are numerous extensions of the model described here, for example one can account for inter-trial variance of *v* and the starting position (Ratcliff and McKoon, 2008), but these parameters can only be reliably estimated with more than 5,000 observations per subject (Voss et al., 2013; Lerche et al., 2016). Models including these parameters do not perform better in terms of parameter estimation when applied to rather few data points (Lerche et al., 2016). Hence we restrict ourselves to this model and include only parameters for which we expect possible effects (see Table 1), *t*_0_, and *s*_*t*_0__, which is the model Lerche et al. (2016) propose to counteract the influence of contaminant RTs.

## Methods

### Participants

Our first sample (*N* = 279, 152 females, 126 males, 1 not reported), Sample A, was part of a larger sample of elementary school children from grades two to four, where subjects that scored lower than 85 in an intelligence test were excluded. Apart from intelligence (*M* = 105.85, *SD* = 11.44) subjects were tested for reading fluency (*M* = 98.39, *SD* = 17.09) and mathematical achievement (*M* = 104.76, *SD* = 14.71). The average age in months was 98.39 (*SD* = 17.09).

Children from grades two and three received three sub-tests of the Intelligence Scale 1-Revision (CFT 1-R; Weiß and Osterland, 2012) (Series Completion, Classification, Matrices). Fourth graders received four sub-tests of the Intelligence Scale 2-Revision (CFT 20-R; Weiß, 2008) (Series Completion, Classification, Matrices, Topologies). Mathematical achievement was assessed using four sub-tests of the Arithmetic Operations of the Heidelberger Numeracy Test (HRT 1–4; Haffner et al., 2005) (Addition, Subtraction, MC, Placeholder Tasks). Reading fluency was assessed using the Salzburger Reading Screening (SLS 1–4; Mayringer and Wimmer, 2003). These test were administered in a classroom setting.

Since Sample A contained too little dyscalculic children in order to test hypotheses specifically about dyscalculia, we augmented it by another sample of second, third and fourth graders, who either were previously diagnosed as dyscalculic or scored below the 16% rank in another standardized mathematical achievement test. The only differences in testing procedure between the two samples were the test setting, here single testing, and the administered intelligence and mathematical achievement tests; WLD sub-scale of WISC-IV (Petermann and Petermann, 2011) and ZAREKI-R (von Aster et al., 2006), respectively. Both mathematical achievement tests cover basic numeric as well as basic arithmetic skills. Both intelligence tests use figural content. All tests (including Sample A) were administered by trained and experienced test administrators and testing sessions lasted for about 60 min.

The union of both samples will be referred to as Sample B. Based on the score in the respective mathematical achievement test, Sample B was split into two groups: **con** including all subjects with a score higher than 85, and **dys** including all subjects with a score of 85 or lower. Group sizes, age, and the results of each group in IQ, mathematics, and reading tests are summarized in Table 2. The lower reading fluency among dyscalculia group should be noted. However, Raddatz et al. (2016) showed that a lower reading fluency does not influence accuracy and RTs in our task selection.

**Table 2:** Group size, mean and standard deviation of pre-study test scores, and age in months by subject group in the combined sample B.

	**con**	**dys**
***N***	**272**	**81**
IQ^†^	106.58 (11.31)	99.69 (10.85)
Math. achievement^†^	107.81 (12.31)	78.89 (4.18)
SLS	100.31 (16.33)	82.07 (14.77)
Age (months)	105.61 (9.89)	111.11 (11.51)

† *Two different tests were used*.

### Materials

Computer based tests were administered on Samsung EEE Netbooks with 19 inch screen diagonal during a separate testing session, which lasted approximately 45 min. Distance from screen to eye was approximately 50 cm. Children had to use the keyboard for their answers. Responses and RTs were recorded. In addition to the two computer based tasks that are analyzed in this article and which were used in the subsequent order, the following tasks were also administered on the same computer in the same session: Dot-enumeration, number transcoding, reaction time, number line estimation, calculation, working memory, and two tasks involving dot sets and numerals at the same time.

In the symbolic MC tasks, two single-digit Arabic numbers aligned horizontally were shown on screen (see Figure 1). Subjects had to decide which of them was numerically larger. Three practice trials and 24 test trials were administered. Numerical distances between the two numerals followed a balanced design with each distance between one and six appearing four times. Each item was preceded by a fixation cross lasting for 500 ms. The restriction to the single-digit range allows for comparison with many existing studies and avoids alternative solution mechanisms. For an overview of the latter see Verguts and De Moor (2005) for example.

The Panamath test (Halberda et al., 2008) was used as a dot set MC task. Two sets of dots (colored yellow and blue) were displayed next to each other on the screen and subjects had to decide as fast as possible without counting which of the sets was larger; see also Mazzocco et al. (2011). Following two practice trials, subjects were given a total of 48 items with four different groups of ratios between the two sets (each being approximately 1.2, 1.4, 1.6, or 2.6). Each ratio group was used twelve times. We used similar ratios to Halberda et al. (2008) and Mazzocco et al. (2011). As in the aforementioned studies, half of the trials were controlled for average dot size, the other half was controlled for total area. The number of dots in each set ranged from 5 to 21, such that subitizing was unlikely (Rousselle and Noël, 2007).

### Data treatment and diffusion model estimation

An inspection of RTs and error rates on item level revealed that the responses to the first symbolic MC item as well as to the first three non-symbolic MC items were very slow; the third non-symbolic item also had an overall error rate above fifty percent. Since item difficulty could not explain these results, all these items were regarded as additional practice trials and excluded from analysis.

Common procedures to remove contaminant RT data fail to remove all contaminant RTs and remove genuine data as well (Lerche et al., 2016). Since we had comparatively little data points for a DM analysis, we had to balance estimation efficiency and robustness against RT outlier. We chose the Kolmogorov-Smirnoff procedure as implemented in fast-dm (Voss and Voss, 2007) for its robustness and fairly high efficiency (Lerche et al., 2016). Another benefit of this procedure is that it does not introduce a bias dependent on the number of observed items (Lerche et al., 2016), i.e., our parameter estimates will not be biased because of the different numbers of items used in symbolic and non-symbolic conditions. The more efficient maximum likelihood estimation introduces such a bias in boundary separation estimates and is sensitive to fast guesses (Lerche et al., 2016). We refrained from explicitly modeling RT outliers (Ratcliff and Tuerlinckx, 2002), since it would introduce new parameters and—given the low amount of data—would not improve parameter estimation (Lerche et al., 2016).

Following recommendations of Lerche et al. (2016) and the procedures of Ratcliff et al. (2012) and Park and Starns (2015), we chose fixed lower and upper cut-offs beyond which responses were discarded. Responses faster than 250 ms (309 responses, 1.01% removed in total) as well as responses slower than four seconds (384 responses, 1.25% removed in total) were removed from analysis. Subjects that gave responses outside these time limits to more than half the items of any tasks were excluded from analysis; one subject was excluded this way.

**Figure 3:**
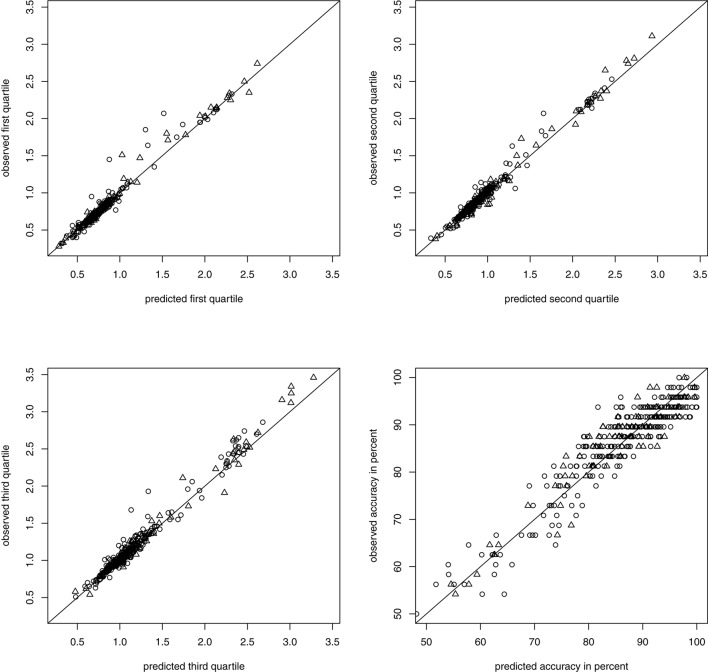
Predicted and observed accuracy and quartiles of RTs in seconds in non-symbolic MC separated by group membership: **con** depicted as circles, **dys** as triangles.

**Figure 4:**
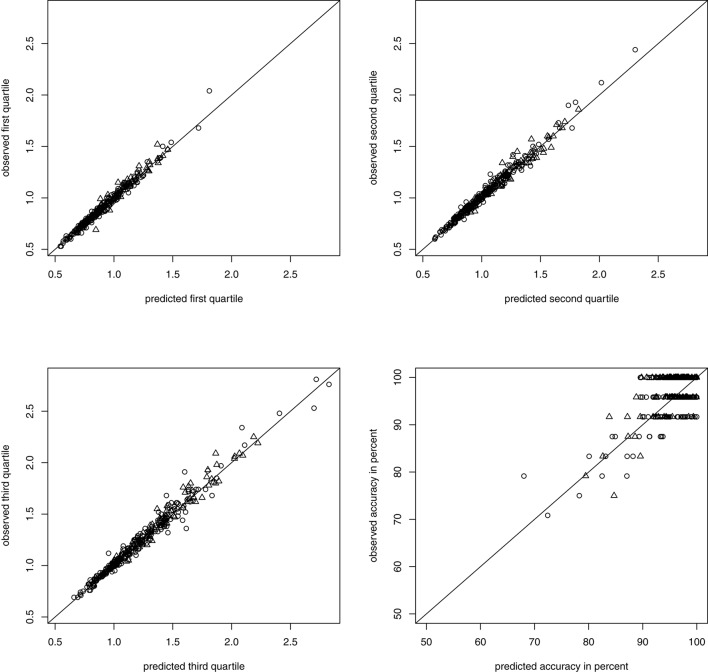
Predicted and observed accuracy and quartiles of RTs in seconds in symbolic MC separated by group membership: **con** depicted as circles, **dys** as triangles.

To assess global model fit, we compared the observed quartiles of the RTs and accuracy for each person with the quartiles and accuracy predicted by the model with the estimated parameters following the graphical procedure described in Voss et al. (2013). Prediction of RTs was very good (non-symbolic) to exceptional (symbolic) while prediction of accuracy was decent (non-symbolic) to fair (symbolic) and there was no systematic bias for the whole sample nor any of the subgroups, con and dys (see Figure 3 for non-symbolic and Figure 4 for symbolic MC).

The results of Lerche et al. (2016) regarding parameter recovery allow for a rough estimation of the reliability of the diffusion model parameters. However, since the reliability depends on the selection of parameters, the parameter values, and model fit, split-half reliability was computed for the diffusion model parameters, boundary separation, mean drift rate, and non-decision time, and the Spearman-Brown corrected results are summarized in Table 3.

**Table 3:** Split-half reliability of boundary separation, mean drift rate, and non-decision time in both types of MC after adjustment using the Spearman-Brown prediction formula.

**Parameter**	**Reliability**	
	**Symbolic MC**	**Non-symbolic MC**
*v*	0.42	0.60
*a*	0.53	0.52
*t*_0_	0.75	0.96

### Descriptive measures

Mean accuracy score and median RT of correctly answered items were computed for every subject and task type. In order to compare our results directly with Park and Starns (2015) Weber fractions were estimated despite their disadvantages compared to other measures (Inglis and Gilmore, 2014). Weber fraction was estimated for each subject within the interval [0, 3] using a maximum likelihood estimator according to the formulae in Halberda et al. (2008). See **Table 5** for a summary of the results.

Weber fraction for non-symbolic MC is correlated with *r*_(277)_ = −0.96 with mean accuracy and thus correlations and effect sizes are virtually identical (albeit with a different sign). As the Bonferroni-Holm adjustment accounts for independent multiple testing, Weber fraction was disregarded in subsequent adjustments.

### Correlations with mathematical achievement

Correlations of DM parameters (*a, v, t*_0_), median RT, and mean accuracy with HRT (mathematical achievement) score in Sample A are displayed in Table 4. After a Bonferroni-Holm adjustment we found significant correlations between mathematical achievement and the following parameters: Median RT, *r*_(277)_ = −0.26, *p* < 0.01, *v*, *r*_(277)_ = 0.25, *p* < 0.01, and *a*, *r*_(277)_ = −0.21, *p* < 0.01, in symbolic MC and mean accuracy, *r*_(277)_ = 0.20, *p* = 0.01, and *v*, *r*_(277)_ = 0.24, *p* < 0.01, in non-symbolic MC. For each measure we compared its correlation with mathematical achievement between both task types using William's test and found a significant difference only for *a*, *t*_(277)_ = −2.23, *p* = 0.03.

**Table 4:** Correlations of dependent measures with HRT (curriculum based mathematical achievement) scores.

**Measure**	**Correlation with HRT**	
	**Symbolic MC**	**Non-symbolic MC**
*v*	**0.25**	**0.24**
*a*	−**0.21**	−0.03
*t*_0_	−0.13	−0.06
*s*_*t*_0__	−0.15	−0.16
Mean accuracy	0.05	**0.20**
Weber fraction	–	−**0.21**
Median RT	−**0.26**	−0.10

*Correlations that are significant (after a Bonferroni-Holm adjustment) are written in boldface*.

### Comparison of dyscalculic and control children

For the group comparison of Sample B Cohen's *d* was computed using the pooled variance. We furthermore computed confidence intervals for *d* and Bonferroni-Holm adjusted *p*-values based on a two sample Welch's *t*-test. These results are summarized in Table 5.

**Table 5:** Means and standard deviations for all task types and measures separated by group.

**Task**	**Measure**	**Con μ (*sd*)**	**Dys μ (*sd*)**	***d***	***CI*_*low*_**	***CI*_*up*_**
Symbolic MC	*v*	2.44 (0.9)	1.92 (0.62)	**0.62**	0.36	0.87
	*a*	1.5 (0.48)	1.73 (0.41)	−**0.49**	−0.75	−0.24
	*t*_0_	0.73 (0.17)	0.78 (0.18)	−0.26	−0.51	−0.01
	*s*_*t*_0__	0.23 (0.22)	0.33 (0.3)	−0.38	−0.63	−0.13
	Mean accuracy	0.97 (0.05)	0.96 (0.05)	0.08	−0.17	0.33
	Median RT	1.00 (0.24)	1.15 (0.25)	−**0.64**	−0.90	−0.39
Non-symbolic MC	*v*	1.68 (0.7)	1.46 (0.69)	0.32	0.07	0.57
	*a*	1.49 (0.47)	1.6 (0.43)	−0.23	−0.48	0.02
	*t*_0_	0.61 (0.33)	0.8 (0.57)	−0.46	−0.72	−0.21
	*s*_*t*_0__	0.26 (0.23)	0.43 (0.43)	−**0.57**	−0.82	−0.31
	Mean accuracy	0.88 (0.1)	0.87 (0.1)	0.10	−0.15	0.35
	Weber fraction	0.77 (0.72)	0.85 (0.76)	−0.11	−0.36	0.14
	Median RT	0.93 (0.36)	1.19 (0.61)	−**0.60**	−0.86	−0.35

*Resulting effect sizes d with corresponding confidence interval. Significant effects (after a Bonferroni-Holm adjustment) are displayed in bold face. Median RT, t_0_, and s_t_0__ are displayed in seconds*.

In symbolic MC the results are comparable to the results of just Sample A, i.e., there is a significant effect on *v*, *t*_(186.65)_ = 5.91, *p* < 0.01, *a*, *t*_(148.32)_ = −4.21, *p* < 0.01, and median RT, *t*_(123.38)_ = −4.89, *p* < 0.01. For non-symbolic MC only the effects on *s*_*t*_0__, *t*_(92.55)_ = −3.25, *p* = 0.01, and median RT, *t*_(95.93)_ = −3.62, *p* < 0.01, remained significant. Effect sizes of each measure did not differ significantly across task types, despite differences in significance of the respective effects themselves. In particular, all effects had the same sign and were significant prior to adjustment except for mean accuracy (Weber fraction) and *a* in non-symbolic MC.

### ANOVA results

**Figure 5:**
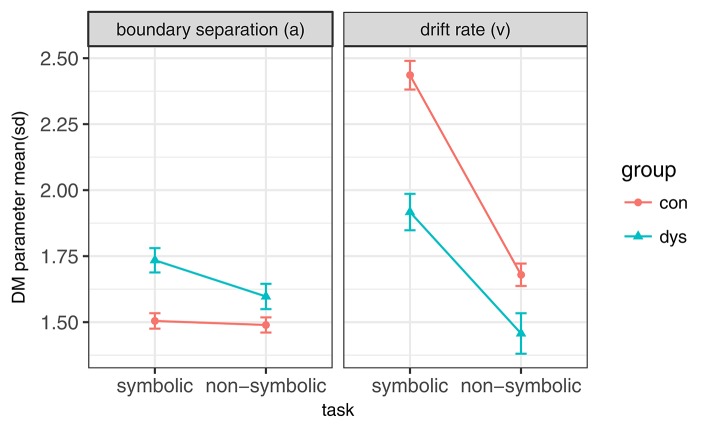
Sample mean and Standard deviation of *v* and *a* in both MC and both subject groups (**con** and **dys**).

A task type (symbolic/non-symbolic) × group (dyscalculic/control) repeated measures ANOVA was conducted on mean drift rate (*v*) and boundary separation (*a*). The dyscalculia group showed significantly lower *v* and higher *a*, we found a significant negative task type × group interaction effect on *v* but no significant interaction on *a*. Significance did not change after a Bonferroni-Holm adjustment. Results are summarized in Figure 5 and Table 6. We also performed analog ANOVAs with accuracy and median RTs as dependent variables and found no task type × group interaction. These results did not change in terms of significance in a linear mixed effects model, where reading fluency was added as predictor for drift rate (respectively boundary separation) above group membership, task type, and their interaction.

**Table 6:** Results of the two-way repeated measures ANOVA of *v* and *a*.

**Parameter**	**Effect**	***F***	**η^2^**	***p*-value**
*v*	DD	23.81	0.039	**<0.01**
	DD × task-type	5.64	0.006	**0.02**
*a*	DD	14.40	0.023	**<0.01**
	DD × task-type	2.43	0.003	0.12

*Significant results (after a Bonferroni-Holm adjustment) are written in bold face*.

## Discussion

In this study we applied a method that has seen little use in the area of DD research, namely diffusion modeling. Alongside the diffusion model results, we analyzed two measures most widely spread in DD research, RTs and accuracy. The data were collected from two samples, one representative of the general population of elementary school children, and one sample suitable for group comparison between DD subjects and healthy controls. These data allowed us to compare our results to results of previous studies, evaluate informational benefits, fit, and requirements of a diffusion model analysis, and answer the question to what extent an impaired ANS and/or an access deficit are associated with DD.

Before we address specific results, let us begin with the technicalities of the diffusion model and the limitations of our study, which arise from these. Our reliability is lower than what could be expected by Lerche et al. (2016) and we would recommend more than one hundred trials even for very basic models if reliability is a concern. Since we used less trials, we obtained attenuated correlations, when diffusion model parameters are involved. This means, correlations close to zero do not necessarily indicate the absence of an association. However, correlations significantly different from zero indicate a considerable effect, that should be investigated in more detail in future studies. Similarly concerning group comparisons, there is more unexplained variance due to unreliability. As such the absence of effects or differences thereof should be viewed with caution, however that doesn't devalue the significant effects we found.

We did not test in terms of significance for model fit, which is a topic on its own (Ranger et al., 2017). However, we did not specify a model that could fit the data in this sense and completely disregarded some aspects, such as fatigue or post error slowing. Instead we chose a simple model that can be estimated with our data and checked whether it predicts observed RTs and accuracy. Our model does this overall well and very well regarding RTs. The fit of accuracy is not as good. However, there is neither an overall bias nor bias on a group level.

Let us begin with the novel results of Sample A, a representative sample in terms of IQ, mathematical ability and reading fluency. We found a positive correlation of drift rate in symbolic MC and mathematics achievement. This means that the ability to compare numerals regarding magnitude is positively related to mathematical achievement. Which was expected but until now only tested using RTs or accuracy as proxies of ability.

We also found a negative correlation of boundary separation in symbolic MC with mathematical achievement, i.e., subjects with low mathematical achievement act more carefully in symbolic MC or in other words they choose higher accuracy over a faster response. Keeping in mind how response behavior and ability interact with RTs and accuracy, our results indicate that RTs in symbolic MC overestimate the deficits of subjects with low mathematical ability, while accuracy underestimates deficits of subjects with low mathematical ability. The higher response caution and the reason for it should be addressed by future studies. It could be an artifact of the test setting or it might be mediated through mathematics anxiety (see Maloney and Beilock, 2012 for an overview), which is present already in first grade and associated with individual mathematical performance (Ramirez et al., 2013).

We further found that correlation of boundary separation and mathematical achievement was significantly higher (closer to zero) for non-symbolic MC than for symbolic MC. This means the contribution of the actual ability toward RTs and accuracy differs between the two task types. This difference renders the test setting as a cause of elevated response caution in symbolic MC less likely. On the other hand the two types of MC differed not only in the form of magnitude presentation but also in the range of magnitudes. However, we chose common ranges for both tasks to allow for comparability with existing research; in fact nearly all studies reviewed by De Smedt et al. (2013) use the 1–9 range for symbolic MC and the overall setup for non-symbolic MC in our study was nearly identical to Halberda et al. (2008) and Mazzocco et al. (2011). The main implication for future research is that neither RTs nor accuracy can be interpreted as ability, when both task types are involved. In particular, existing comparisons between the two tasks types should be reevaluated.

In terms of existing results, we found a negative correlation of median RTs in symbolic MC with mathematics achievement and a slightly weaker correlation of mean accuracy in non-symbolic MC with mathematics achievement. These results are perfectly in line with the meta-analysis by Schneider et al. (2016). Furthermore regarding non-symbolic MC we found a significant correlation between drift rate and mathematics achievement. In conjunction with the absence of a correlation between boundary separation and the latter our results replicate the findings of Park and Starns (2015) albeit with a younger sample.

In Sample B, which contained Sample A as a subsample, we directly compared subjects with and without DD. In the ANOVA we found that DD children had a significantly lower drift rate overall. Following the assumption of Rousselle and Noël (2007) that the ANS is used in both tasks, this indicates that DD subjects suffer from an impaired ANS.

Furthermore the significant negative interaction of symbolic task type and DD on drift rate means that DD subjects have an even lower ability in symbolic MC (compared to healthy children). Still following Rousselle and Noël (2007), this indicates that DD subjects suffer from an access deficit. In this context our findings support the conjoint hypothesis of an impaired ANS and access deficit in DD children. However, scores in symbolic MC might alternatively reflect the symbolic representations themselves (De Smedt et al., 2013). If the ANS was not involved in symbolic MC, we could still conclude, that the ability in symbolic MC was more discriminative in the assessment of DD than the ability in non-symbolic MC. Which in turn would imply that the ANS was less relevant.

Contrary to our expectation we found an overall difference in response caution between healthy and dyscalculic children in the ANOVA. Since all computer based testing occurred at the university, the testing environment might have intimidated dyscalculic children (more than healthy children).

We also found a higher variability of non-decision time in DD children. This could at least be partly attributed to attention problems which are associated with DD (Shalev et al., 1995) or lower mathematical ability (Tosto et al., 2015). Additionally, the difference in IQ scores between the two groups should be noted. We cannot account for IQ the way we could for reading fluency, since two different tests were used with varying proportions in the two groups. This means the difference of IQ scores can be caused by the difference between the tests, the test settings, or subjects. A difference between subjects regarding IQ likely implies a difference between the diffusion model parameters and thus should be as well considered as cause of the latter. However, since no subject with IQ below 85 was included, our results remain valid without limitations for the practical assessment and treatment of DD.

We found that Dyscalculics were significantly slower in both MC tasks, which is in line with current literature (De Smedt et al., 2013). We did not find a significant effect on accuracy or weber fraction for non-symbolic MC. However, four out of six studies reviewed by De Smedt et al. (2013) that report accuracy or weber fraction do not find a significant effect either. This is in contrast to studies regarding the general association of mathematical achievement and performance in non-symbolic MC, where accuracy and mathematical achievement are consistently associated (De Smedt et al., 2013; Schneider et al., 2016).

In summary superficial differences in RTs and accuracy between healthy and DD children (or children with lower mathematical ability) across MC tasks can not be solely attributed to differences in ability but should also be attributed to differences in response behavior. As a conclusion future research should not only account for cognitive confounders like attention problems but also for possible behavioral confounders like mathematics anxiety or better assess response behavior.

Despite the limitations of our study, which are primarily the small number of symbolic MC items and the omission of the aforementioned confounders, we could demonstrate the merits of a diffusion model analysis for DD research. By using this approach we could show that both ANS and an access deficit play a role in DD and we found that response behavior influences the performance and does so to varying degree depending on the mode of stimulus presentation. All of which are relevant not only for future research in numerical cognition and but also for assessment and treatment of Dyscalculia.

## Ethics statement

The legal guardian of each participant provided written informed consent. The study and consent procedure was approved by the Ethikkommission des Fachbereichs Psychologie und Sportwissenschaften of the Westfälische Wilhelms-Universität.

## Author contributions

CS wrote the manuscript and conducted the statistical analysis. J-TK conducted the study. JR provided advice on the statistical analysis and revised the manuscript. HH provided advice on the manuscript.

### Conflict of interest statement

The authors declare that the research was conducted in the absence of any commercial or financial relationships that could be construed as a potential conflict of interest.
